# Aquaglyceroporins Are Differentially Expressed in Beige and White Adipocytes

**DOI:** 10.3390/ijms21020610

**Published:** 2020-01-17

**Authors:** Inês Vieira da Silva, Francisco Díaz-Sáez, António Zorzano, Anna Gumà, Marta Camps, Graça Soveral

**Affiliations:** 1 Research Institute for Medicines (iMed.ULisboa), Faculty of Pharmacy, Universidade de Lisboa, 1649-003 Lisboa, Portugal; imvsilva@ff.ul.pt; 2 Department of Biochemistry and Human Biology, Faculty of Pharmacy, Universidade de Lisboa, 1649-003 Lisboa, Portugal; 3 Department of Biochemistry and Molecular Biomedicine, Faculty of Biology, Institute of Biomedicine of the University of Barcelona, 08028 Barcelona, Spain; frandiazsaez@gmail.com (F.D.-S.); antonio.zorzano@irbbarcelona.org (A.Z.); aguma@ub.edu (A.G.); 4 CIBER de Diabetes y Enfermedades Metabólicas Asociadas, Instituto de Salud Carlos III, 8029 Madrid, Spain; 5 Institute for Research in Biomedicine (IRB Barcelona), 08028 Barcelona, Spain

**Keywords:** aquaporin, adipocytes, browning, obesity, metabolic diseases

## Abstract

Browning of white adipocytes has been proposed as a powerful strategy to overcome metabolic complications, since brown adipocytes are more catabolic, expending energy as a heat form. However, the biological pathways involved in the browning process are still unclear. Aquaglyceroporins are a sub-class of aquaporin water channels that also permeate glycerol and are involved in body energy homeostasis. In the adipose tissue, aquaporin-7 (AQP7) is the most representative isoform, being crucial for white adipocyte fully differentiation and glycerol metabolism. The altered expression of AQP7 is involved in the onset of obesity and metabolic disorders. Herein, we investigated if aquaglyceroporins are implicated in beige adipocyte differentiation, similar to white cells. Thus, we optimized a protocol of murine 3T3-L1 preadipocytes browning that displayed increased beige and decreased white adipose tissue features at both gene and protein levels and evaluated aquaporin expression patterns along the differentiation process together with cellular lipid content. Our results revealed that AQP7 and aquaporin-9 (AQP9) expression was downregulated throughout beige adipocyte differentiation compared to white differentiation, which may be related to the beige physiological role of heat production from oxidative metabolism, contrasting with the anabolic/catabolic lipid metabolism requiring glycerol gateways occurring in white adipose cells.

## 1. Introduction

Adipose tissue has a central role in the regulation of energy homeostasis through its metabolic and endocrine functions, and alterations in its physiological functions associated with a sedentary life and saturated fat-based diets have been related with the development of obesity [1,2]. However, not the whole adipose tissue has the same structure and functions. White adipose tissue (WAT) is an anabolic tissue involved in energy storage in the triacylglycerol form, contrasting with brown adipose tissue (BAT) that is very catabolic and involved in body thermogenesis [3]. Since the accumulation of excess WAT has deleterious consequences for metabolic health and the activation of BAT confers beneficial effects on adiposity, browning the white adipose tissue has been described as a potential strategy to target and control obesity.

Considering that the amount of metabolically active BAT is particularly low in human adults and consequently in obese and diabetic patients who require immediate therapy, new strategies to increase the capacity for adaptive thermogenesis are paramount. Recent findings showed that, in human adults, BAT might consist of not only classic brown adipocytes, cells that originate from myogenic lineage, but also inducible brown adipocytes (also called beige, white-in-brown, or brite adipocytes), which are phenotypically distinct from both white and brown adipocytes but have origin from mesenchymal precursors, such as white adipocytes, and can differentiate from them [4,5]. Browning the white adipocytes gives origin to a third type of adipocyte, beige adipocytes, more frequently found in subcutaneous WAT. Similar to brown adipocytes, beige adipocytes have multiple lipid droplets and UCP1-rich mitochondria and can acquire thermogenic features. On the other hand, comparable to white adipocytes, they can trigger the storage phenotype, depending on the surrounding environment [6].

Stimulating the development of beige adipocytes in WAT (so called “browning”) might reduce adverse effects of WAT and can help to improve metabolic health [7]. Despite their different origins, beige and brown adipocytes show increased expression of UCP1 and decreased expression of leptin through adrenergic stimulation compared to white adipocytes [8]. BAT activation has also been related to adrenoreceptor stimulation in response to cold temperatures which is translated in increased levels of UPC1 and in browning of white adipocytes [9,10]. Nowadays, it is generally accepted that exercise also triggers browning [11]. Altogether, this data suggests that browning can protect against obesity and metabolic-related complications. Nevertheless, there is still a lack of knowledge of the mechanisms involved in the browning process that lead to differentiation of beige adipocytes.

Aquaporins are integral membrane proteins that function as channels, permeating water and small solutes across biological membranes driven by osmotic or solute gradients [12,13]. Currently there are thirteen isoforms described in humans (AQP0–AQP12) that are widely distributed among the body and differentially expressed in different tissues, playing an important role in a variety of physiological roles [14]. This family of proteins has been distributed by three subgroups according to their selectivity and primary structure: Classical/orthodox aquaporins (AQP0, AQP1, AQP2, AQP4, AQP5, AQP6, and AQP8) considered primarily selective to water, aquaglyceroporins (AQP3, AQP7, AQP9, and AQP10) that also permeate glycerol, urea, and other small noncharged solutes, and nonorthodox/S-aquaporins (AQP11 and AQP12) comprising intracellular isoforms whose selectivity is still under investigation [15,16,17]. Recently, a few isoforms have also been reported to transport hydrogen peroxide and were termed peroxiporins (AQP3, AQP5, AQP8, and AQP9) [18,19,20,21,22].

The aquaglyceroporins, as facilitators of glycerol membrane permeation, are tightly involved in glycerol metabolism and homeostasis, being crucial for energy production in different organs (liver, adipose tissue, and muscle) and with implications in obesity and metabolic-related complications, such as type-2 diabetes and insulin resistance [23,24,25,26,27]. Thus, we hypothesized that aquaglyceroporins are important interveners in beige adipocytes genesis. Moreover, our group previously reported an essential role for AQP5 and AQP7 in white adipocytes differentiation. AQP7 increases gradually along with the progression of the mature white adipocyte phenotype whereas AQP5 seems to be essential for the differentiation to occur [28,29].

In this study, we used the well-characterized murine 3T3-L1 preadipocyte cell line to optimize a good and feasible protocol of beige adipocyte differentiation in comparison with white adipocyte differentiation, while evaluating the expression of biomarkers of beige/brown (UCP1, CD137, TBX15, TBX1, TIMM44, and NRG4) and white (LPL and GLUT4) differentiation. Once achieved, the beige adipocyte phenotype, the expression level of AQP3, AQP7, AQP9, and AQP5 together with cellular triacylglycerol content was investigated throughout the differentiation process until cells were fully differentiated. Moreover, aquaporin expression pattern was correlated with biomarkers of beige differentiation during browning.

## 2. Results

### 2.1. Induction of Beige Adipocyte Phenotype in 3T3-L1 Preadipocytes

**Figure 1:**
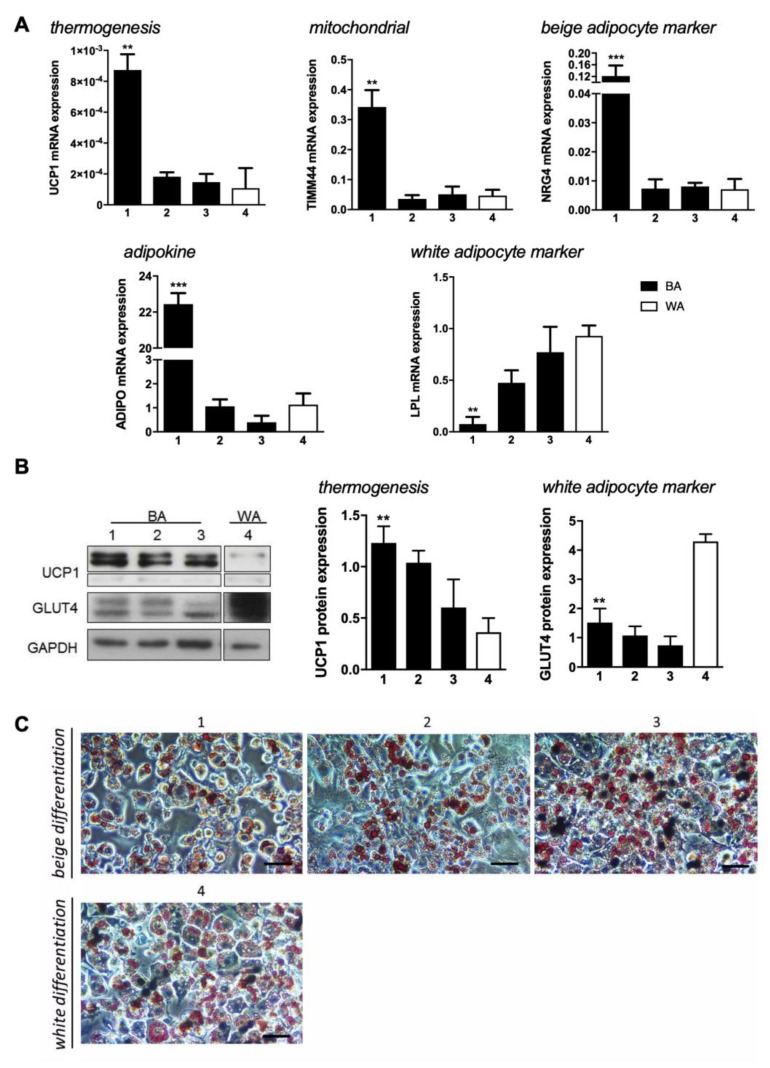
Beige and white adipocyte markers expression and their modulation by four differentiation-inducing cocktails in 3T3-L1 cells. (**A**) Relative expression of UCP1, TIMM44, NRG4, ADIPO, and LPL in 3T3-L1 fibroblasts that were induced to differentiate into beige (1–3; black bars) (BA) and white (4; white bars) (WA) adipocytes. Gene expression values are relative to ARP. (**B**) Representative blots and relative UCP1 and GLUT4 protein expression in 3T3-L1 fibroblasts that were induced to differentiate into beige (1–3; black bars) and white (4; white bars) adipocytes in relation to Glyceraldehyde 3-phosphate dehydrogenase (GAPDH). (**C**) Lipid droplets characterization by triacyclglycerols staining with Oil Red *O* expression in 3T3-L1 fibroblasts that were induced to differentiate into beige and white adipocytes. Scale bar = 20 µm. Data represent mean ± SEM of three independent experiments. * *p* < 0.05, ** *p* < 0.01, *** *p* < 0.001.

To achieve the beige adipocyte phenotype, three induction cocktails for browning and one for white cells differentiation were tested in 3T3-L1 preadipocytes (Figure 1). 3T3-L1 cells were induced to differentiate by treatments with triiodothyronine (T3) (protocol 1 and 3), L-rhamnose (protocol 2), and/or salicylate (protocol 3) in addition to the reagents used in the control protocol (insulin, IBMX, rosiglitazone, and dexamethasone) that targets the white adipocyte phenotype (protocol 4). Subsequently, the more feasible protocol of “browning” was ascertained by evaluating gene expression of several brown, beige, and white adipocyte markers.

Our data reveal that the mitochondrial uncoupling protein 1 (UCP1) responsible for dissipation of the proton gradient generated by oxidative phosphorylation producing heat, is higher in browning protocols (1–3; black bars), compared to control (4; white bar). In addition, protocol 1 is seen to induce significantly higher UCP1 gene expression. The mitochondrial marker TIMM44 is also seen to be expressed at higher levels in protocol 1, suggesting higher mitochondrial mass or more active mitochondria in beige cells. The beige marker NRG4 [30] is significantly increased in cells differentiated using protocol 1, compared to protocols 2–3. The evaluation of ADIPO expression showed that this cytokine, that is mainly produced by adipocytes, is also upregulated by the protocol 1 treatment. On the other hand, LPL, a membrane enzyme essential for the hydrolysis of triacylglyceride and lipoprotein-associated fatty acids, is expressed in much lower amounts in protocol 1 than in the other protocols, confirming distinct beige features (Figure 1A).

At the protein level, UCP1 data are in accordance with the gene expression, where protocol 1 seems to be the one that induces higher production of this protein. GLUT4, the insulin-dependent glucose transporter that is highly expressed in white adipocytes during the feeding state, showed to be less expressed by any browning protocol than by protocol 4 (Figure 1B). In addition, staining 3T3-L1 cells at day 7 of differentiation with Oil Red *O* disclosed that all protocols were successful for adipocyte differentiation regardless the treatment (Figure 1C) since all treatments resulted in triacylglycerol-rich lipid droplets. However, protocols 1 and 2 promoted the development of smaller lipid droplets and in lower number. Our data suggests protocol 1 as the most efficient in inducing a beige phenotype compared to the control white phenotype (protocol 4).

### 2.2. Aquaglyceroporins are Differentially Expressed in Beige and White Adipocytes

**Figure 2:**
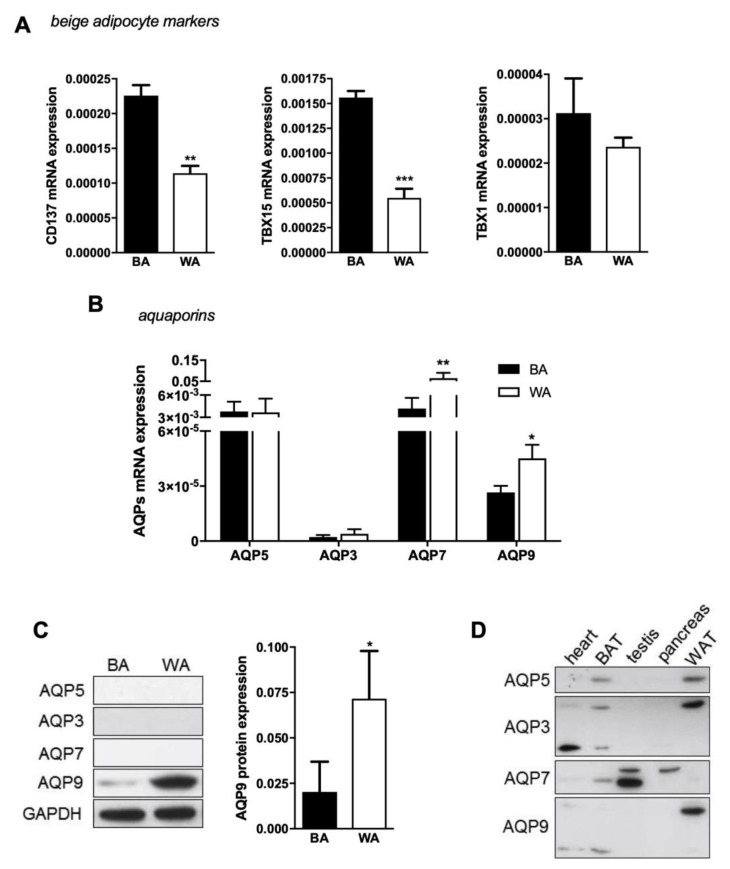
AQP7 and AQP9 differential expression in beige and white adipocytes. (**A**) Relative expression of several beige adipocyte markers (CD137, TBX15, and TBX1) and (**B**) aquaporins (AQP5, AQP3, AQP7, and AQP9) in 3T3-L1 fibroblasts induced to differentiate into beige (BA; black bars) and white (WA; white bars) adipocytes. Gene expression values are relative to ARP and to Eef2. (**C**) Representative blots and relative AQP9 protein expression in 3T3-L1 fibroblasts that were induced to differentiate into beige (BA; black bars) and white (WA; white bars) adipocytes in relation to GAPDH. A single band of 33 kDa was detected in cultured cells. (**D**) Antibodies validation against AQP5, AQP3, AQP7, and AQP9 in murine tissues: Heart, BAT, testis, pancreas, and WAT. Data represent mean ± SEM of three independent experiments. * *p* < 0.05, ** *p* < 0.01; *** *p* < 0.001, beige vs. white adipocytes.

After choosing protocol 1 as the best browning protocol to produce beige adipocytes, other few beige markers were also evaluated by qPCR (Figure 2A). Although no significant differences were observed for TBX1 expression when using different protocols, we found CD137 and TBX15 genes to be expressed at higher levels in beige (BA) than white adipocytes (WA) (Figure 2A), in accordance with the above results and validating the browning process. Then, we investigated the aquaglyceroporins AQP3, AQP7, and AQP9 and the orthodox AQP5 gene expression levels in cells resulting from beige and white differentiation.

The four investigated isoforms are differentially expressed in beige and white adipocytes. AQP5 and AQP7 are the most representative aquaporins in beige cells and are expressed in similar levels, followed by AQP9 and AQP3 in lower levels (Figure 2B; black bars). In white cells, AQP7 is the most expressed isoform, followed by AQP5, AQP9, and AQP3 (Figure 2B; white bar). However, at the protein level, only AQP9 expression was detected in beige adipocytes and its abundance was lower than in white adipocytes (Figure 2C). AQP9 protein expression is in accordance with its gene expression level and is significantly lower in beige than in white cells. Interestingly, AQP7 protein was not detected in white and in beige as expected. Therefore, we proceeded to antibodies validation against AQP3, AQP5, AQP7, and AQP9 in several murine tissues, such as heart, BAT, testis, liver, pancreas, and WAT (Figure 2D). As depicted, all antibodies detected AQPs in the different tissues, and in addition, aquaglyceroporins were detected with different levels of glycosylation (Figure 2D), a condition that has already been reported for several isoforms [31].

### 2.3. Beige Adipose Cell Differentiation Is a Late Event

**Figure 3:**
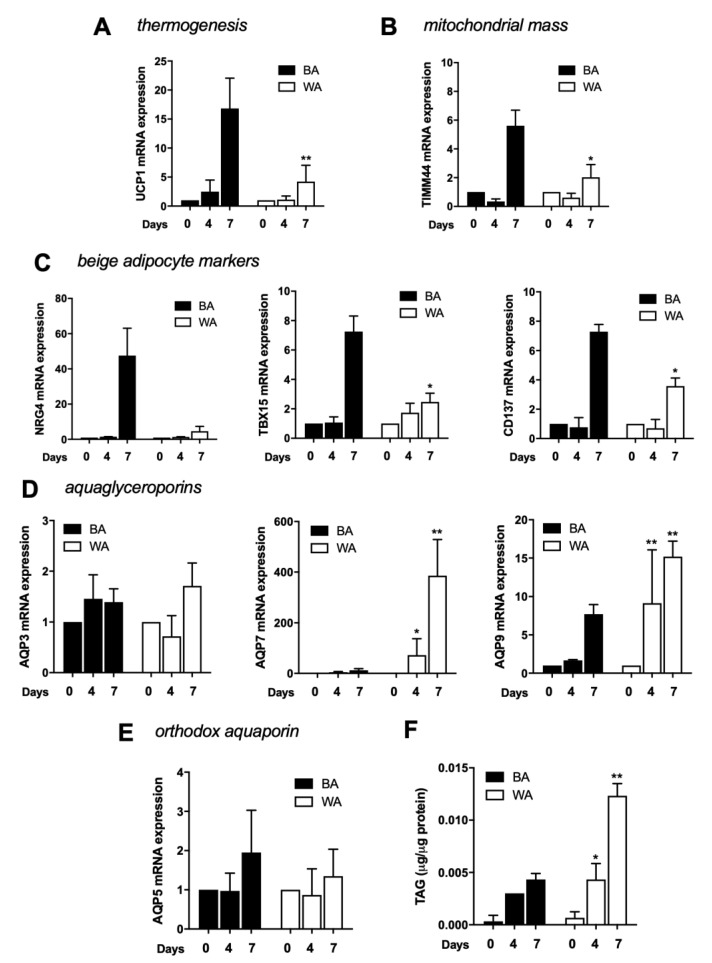
Beige phenotype achievement along differentiation. (**A**) Relative expression of UCP1, (**B**) TIMM44, (**C**) NRG4, TBX15, CD137, (**D**) AQP3, AQP7, and AQP9 and (**E**) AQP5, in 3T3-L1 fibroblasts induced to differentiate into beige (BA; black bars) and white (WA; white bars) adipocytes at day 0, 4, and 7 of differentiation. Gene expression values are relative to ARP and Eef2 and to day 0. (**F**) Triacylglycerols accumulation in 3T3-L1 fibroblasts induced to differentiate into beige (BA; black bars) and white (WA; white bars) adipocytes at day 0, 4, and 7 of differentiation. Data represent mean ± SEM of three independent experiments. * *p* < 0.05, ** *p* < 0.01, differentiation time point vs. previous time point.

Since the induction cocktails for differentiation in beige and white adipocytes are very similar, both consisting in insulin, rosiglitazone, IBMX, and dexamethasone, with the increment of T3 for beige differentiation, we evaluated several beige markers and aquaporin gene expression along the differentiation process, specifically at day 0, 4, and 7 (Figure 3).

Beige cells showed a significant increase of the thermogenic marker UCP1 at day 7 of differentiation (Figure 3A). Similarly, the mitochondrial mass marker TIMM44 showed the same dynamics (Figure 3B). NRG4, TBX15, and CD137, were evaluated as beige adipocyte markers and were also shown to be drastically increased at day 7 (Figure 3C). When evaluating aquaporins expression during differentiation, both AQP7 and AQP9 are found upregulated, but while AQP7 increases gradually, AQP9 transcript increases abruptly at day 7 (Figure 3D). AQP3 and AQP5 seem to be constitutively expressed during the process (Figure 3D,E). The quantification of intracellular triacylglycerols in beige cells showed an evident increase in TAG content until day 4 (Figure 3F).

The same analysis was performed for cells induced to differentiate in white adipocytes. As expected, beige markers are less expressed in white cells. While AQP3 and AQP5 expression are not affected by differentiation and show similar expression levels in WA and BA, AQP7 and AQP9 expression level dramatically increases during differentiation showing higher levels of transcript in WA (Figure 3), as previously reported [28].

## 3. Discussion

In the last decades, increasing BAT has emerged as a potential solution for obesity and type-2 diabetes [32,33]. In humans, the amount of BAT is low and it reduces drastically in the first years of age [34]. Browning the white adipocytes by inducing cells to acquire a phenotype more like brown cells, was recently reported [35], and the advantage of the thermogenic features of these cells has become an experimental strategy to burn excess of WAT-stored triacylglycerols, opening new perspectives for obesity, and excess fat-related complications therapies. AQPs, namely aquaglyceroporins, are transmembrane channels very well documented as important players in energy homeostasis control. In fact, although all aquaglyceroporins (AQP3, 7, 9, and 10) are expressed in human adipose tissue, AQP7 expression increases along with white adipocyte differentiation and has been reported to be involved in obesity [26,27,28,36,37]. Beige adipocytes, differentiated from white cells, are part of a recent strategy for obesity therapy because of their thermogenic features instead of storage ones [11,35]. Therefore, after evaluating several browning protocols, we used the one that best fits to trigger the beige adipocyte phenotype to investigate AQP3, AQP5, AQP7, and AQP9 differential expression in beige and white adipocytes differentiated from murine 3T3-L1 preadipocytes.

Insulin, dexamethasone, rosiglitazone, and IBMX are commonly used to differentiate 3T3-L1 preadipocytes in white adipocytic cells. Although rosiglitazone has been reported to have browning effects during the adipocyte by activating MAPK and PI3K signalling pathways [38], it has also been extensively reported as an enhancer of white adipocyte full differentiation since it triggers peroxisome proliferator activator receptor gamma (PPARγ) overexpression, the master regulator of both BAT and WAT adipogenesis [39,40]. Adding factors that target β-adrenergic stimulation, such as L-rhamnose, T3, irisin, fibroblast growth factor 21, or follistatin to the previous cocktail have been described to induce fat browning [32,41,42,43,44]. Protocol 1 which consisted in T3 treatment, in addition to the basal cocktail for white adipocyte differentiation, showed to be the most successful in inducing beige phenotype mainly because this thyroid hormone potentiates the effects of the β-adrenergic receptors in glucose metabolism and increases the thermogenic capacity of adipose tissue to enhance energy expenditure [45]. Our data revealed an upregulation of brown/beige-related markers such as UCP1, TIMM44, NRG4, TBX15, CD137, and ADIPO gene expression in cells treated with protocol 1 compared to control, while LPL gene expression was decreased. At the protein level, UCP1 results are in agreement with the found at the transcript level, whereas GLUT4 showed lower expression in browning protocol than control. Previous experiments performed in immortalized cell lines from different adipose tissue depots described higher levels of GLUT4 transcript in BAT-derived cell lines compared to WAT-derived cell lines [46]; however, no descriptions on GLUT4 levels in beige adipocytes compared to white adipocytes have been reported. In addition, preadipocytes induced to differentiate in beige cells revealed less triacylglycerol accumulation. T3 is one of the hormones produced in thyroid that influences BAT gene expression [45]. It promotes mitogenesis, induces the expression of UCP1 increasing thermogenesis activity and activates brown metabolism [47]. Similarly to BAT, T3 has also been implicated in the induction of the browning process in humans [48].

From our knowledge, this is the first study reporting aquaporins expression in beige adipocytes and comparing their level of expression with white adipocytes. The four screened isoforms (orthodox AQP5 and aquaglyceroporins AQP3, AQP7, and AQP9) are differentially expressed in the two types of cells. The gene expression analysis showed that AQP5 and AQP7 are the most representative aquaporins in beige cells, followed by AQP9 and AQP3 in lower amounts, while in white cells, AQP7 is the most expressed isoform, followed by AQP5, AQP9, and AQP3, in accordance to previous data [28]. However, at the protein level, only AQP9 was detected in beige adipocytes and with lower transcript level than in white cells. The possible effect of differentiation cocktail components, such as T3, on a specific AQP protein synthesis and stability and subsequent downregulation cannot be disregarded.

Along the beige differentiation, a stronger beige-engagement was detected in the last days, from 4 to 7, where UCP1, TIMM44, NRG4, TBX15, and CD137 were drastically increased. Aquaporins gene expression analysis along differentiation shows that AQP7 and AQP9 are upregulated while AQP3 and AQP5 seem to be constitutively expressed during the process. Intracellular triacylglycerols quantification showed a slight increase in TAG content in beige cells compared to white cells.

Overall, our study identifies downregulation of the global aquaglyceroporins expression induced by browning, probably because beige cells are more committed to oxidize and burn fat to produce heat than storing or hydrolyzing lipids and exporting glycerol through an aquaglyceroporin gateway driven by energy unbalance. Our results support the thermogenic characteristics of these interchangeable cells, highlighting the browning strategy as a potential tool for obesity therapeutics.

## 4. Materials and Methods

### 4.1. 3T3-L1 Cell Culture and Treatments

3T3-L1 preadipocytes (CCL 92.1; American Type Culture Collection, Manassas, VA) were induced to differentiate into beige and white adipocytes. 3T3-L1 fibroblasts were grown in 6-well plates until reaching confluence in 10% activated calf serum (CS) (Gibco, Tavarnuzze, Italy) high glucose DMEM (Biowest, Nuaillé, France) with 100 U/mL of penicillin/streptomycin at 37 °C and 8% CO_2_. For beige differentiation, three protocols were tested in order to select the most suitable. For the first protocol (protocol 1), adapted from [41], beige differentiation was induced by adding 0.25 μM dexamethasone (Sigma-Aldrich, St. Louis, MO, USA), 0.5 mM 3-isobutil-1-metilxantina (IBMX) (Sigma-Aldrich, St. Louis, MO, USA), 10 µg/mL insulin (Roche, Basel, Switzerland), and 50 nM triiodothyronine (T3) (Sigma-Aldrich, St. Louis, MO, USA) in 10% (*v*/*v*) fetal bovine serum (FBS) (Gibco) high glucose DMEM (complete medium-CM) for two days, then, 5 µg/mL insulin, 1 µM rosiglitazone, 50 nM T3, and 0.5 mM IBMX in CM for four days, and finally, 1 µM rosiglitazone, 50 nM T3, and 0.5 mM IBMX in CM for two days. Cells were treated with 10 µM isoproterenol for 4 h before harvest. For the second protocol (protocol 2), adapted from [42], beige differentiation was induced by 0.25 µM dexamethasone, 0.5 mM IBMX, 10 µg/mL insulin, and 100 µM *L*-rhamnose (Sigma-Aldrich, St. Louis, MO, USA) in CM for seven days. For the third protocol (protocol 3), adapted from [49], cells were treated with 0.5 μM rosiglitazone, 0.5 mM IBMX, 2 μg/mL dexamethasone, 125 μM salicylate, 5 μg/mL insulin, and 1 μM T3 in CM for two days. Then, cells were treated with 5 μg/mL insulin, 1 μM T3, and 0.5 μM rosiglitazone in CM for two days, and, 5 μg/mL insulin, 1 μM T3, and 1 μM rosiglitazone in CM for three days. Before harvest, cells were treated with 10 μM IBMX for 4 h to induce cAMP. White differentiation (protocol 4) was induced by adding 1 μg/mL insulin, 0.25 μM dexamethasone, 1 μM rosiglitazone, and 0.5 mM IBMX in CM for three days [50]. Afterwards, 1 μg/mL insulin in CM for two days, and finally, cells were incubated with CM for three days. For beige differentiation protocol validation, cells were harvested at day 7 of differentiation, whereas for differentiation studies, cells were harvested at day 0, 4, and 7 of differentiation.

### 4.2. RNA Extraction, Reverse-Transcription, and Quantitative PCR

Total RNA was extracted from 3T3-L1 cells with PureLink™ RNA mini kit columns following the manufacturer’s instructions (Invitrogen, Waltham, MA, USA). RNA was quantified using the Nanodrop™ 2000/2000c spectrometer (Thermo Scientific, Waltham, MA, USA) and the ND1000 software (Thermo Scientific, Waltham, MA, USA). Reverse-transcription was performed by mixing 1 µg RNA with 1 μL 10 mM deoxynucleotide mix and 1 μL 50 μM oligodTs. Samples were heated to 65 °C for 5 min using the 2720™ Thermal Cycler (Applied Biosystems, Foster City, CA, USA) and SuperScript™ solution, prepared by mixing 2 μL of 40 U/μL RNAseOUT™ solution, 4 μL of 5X First-Strand buffer and 1 μL of 200 U/μL SuperScript™ II reverse transcriptase (Invitrogen, Waltham, MA, USA). The obtained cDNA was stored at −20 °C until used. For qPCR analyses, the reaction mix was prepared by mixing 5 μL of SYBR^®^ Green PCR master mix, 0.6 μL of 10 μM primer mix and 0.4 μL of Milli-Q H_2_O, or by mixing 5.5 μL of Taqman^®^ Universal master mix with UNG and 0.5 μL of Taqman probe and primers mix per each sample containing 4 μL of cDNA. Sequence for designed primers (Sigma-Aldrich, St. Louis, MO, USA) are listed in Table 1. Taqman probes and primers used are the following: AQP3 (#Mm01208559_m1), AQP5 (#Mm00437578_m1), AQP7 (#Mm00431839_m10), AQP9 (#Mm00508094_m1), and Eef2 (Mm00833287_g1) (Applied Biosystems, Waltham, MA). qPCR negative controls were loaded to identify cross-contamination. qPCR was performed using the ABI Prism 7900 HT qPCR machine (Applied Biosystems) and the SDS software (Applied Biosystems). Gene expression measurements were normalized to ARP (for designed primers) or Eef2 (for Taqman primers) cDNA using the 2^−ΔΔ*C*t^ method.

### 4.3. Protein Extraction and Western Blotting

Total 3T3-L1 homogenates were washed with cold PBS, scraped in lysis buffer (20 mM Tris-HCl (pH7.5), 150 mM NaCl, 1mM EDTA, 1mM EGTA, 1% (*v*/*v*) NP-40, and 1% (*w*/*v*) sodium deoxycholate) supplemented with protease inhibitor cocktail and homogenized with a syringe. Homogenates were centrifuged at 13200 rpm for 30 min at 4 °C and protein lysates were quantified with Pierce™ BCA protein assay kit (Thermo Scientifics, Waltham, MA) and 20/40 µg of total proteins for target proteins, and 5 µg for internal control were mixed with 4× Laemmli buffer and then boiled 5 min at 95 °C. SDS-polyacrylamide gel electrophoresis was performed and proteins were transferred to PVDF membranes (MERCK Millipore, Darmstadt, Germany). Then, membranes were blocked with 5% (*w*/*v*) nonfat dry milk and incubated overnight at 4 °C with antibodies against GLUT4 (1/1000; produced in the lab by Dr. Conchi Mora [51]), UCP1 (1/1000; Abcam, Cambridge, UK), AQP3 (1/200), AQP5 (1/200), AQP7 (1/200), AQP9 (1/200) (antibodies against AQPs-Santa Cruz Biotechnologies, Dallas, Texas), and GAPDH (1/1000; Cell Signaling, Danvers, MA). All antibodies were diluted in 3% (*w*/*v*) bovine serum albumin, 0.02% (*w*/*v*) sodium azide in TBS-T. On the following day, membranes were incubated for 1 h with HRP-conjugated anti-mouse, anti-rabbit, or anti-goat secondary antibodies (Jackson ImmunoResearch, Cambridgeshire, UK) diluted 1/10,000 in 5% (*w*/*v*) nonfat dry milk in TBS-T. Finally, membranes were incubated with the ECL western blotting detection reagent enhanced chemiluminescence solution to detect specific proteins (GE Healthcare Amersham, Buckinghamshire, UK). The autoradiograms were quantified using ImageJ software.

Validation of anti-AQPs antibodies was performed by immunoblotting using murine tissues (heart, BAT, testis, pancreas, and WAT). Murine tissues were harvested and placed in PBS plus Complete Protease Inhibitor (Roche, Basel, Switzerland) on ice, homogenized for 20 strokes in a drill press, then spun at 1000× *g* for 10 min at 4 °C. The protocol was conducted according to the European Guidelines for the Care and Use of Laboratory Animals (Directive 86/609) and approved by the University of Barcelona Committee on Animal Care.

### 4.4. Oil Red O Staining

Oil Red *O* staining was used to stain neutral lipid inside the cells. For this, preadipocytes/adipocytes were washed twice with PBS and then fixed with 3% paraformaldehyde (PFA) for 15 min at room temperature. The PFA was removed and cells washed with 60% isopropanol. The cells were then incubated with working solution (60% 8.5 mM Oil Red O; 40% H_2_O Milli-Q) previously filtered, for 10 min at 37 °C. Working solution was removed and cells washed three times with H_2_O Milli-Q. Cells were observed and images were registered in bright field. Images were captured using a Motic digital camera and Motic software (Motic, Canada).

### 4.5. Triacylglycerols Quantification

Triacylglycerols quantification was done in total lysates using the Biosystems Triglyceride assay kit. Preadipocytes/adipocytes were lysed using a homogenization buffer (0.25 M Sucrose; 2 mM EGTA; 20 mM HEPES; pH7.4) with protease inhibitors (1 µg/mL Pepstatin; 1 µg/mL Leupeptin; 0.5 mM PMSF; 10 µg/mL Aprotinin). A standard curve from 0 to 200 mg/dL was generated loading serial dilutions of the 200 mg/dL triglycerides standard solution (included in the kit) into the ELISA plate and 10 µL of the lysate were used in the quantification. Then, 200 µL of reactive solution were added to each well and plates were incubated for 5 min at 37 °C. After incubation, plates were read at 500 nm.

### 4.6. Statistical Analysis

Data are showed as mean ± SEM. For statistical studies, the t-student test and one-way analysis of variance (ANOVA) with Tukey’s honest significant difference post-hoc test were used. *p* < 0.05 was considered significant. Data were analyzed using the GraphPad Prism 6 software.

## Figures and Tables

**Table 1:** Gene-specific primer sequences used for real-time-quantitative PCR.

Gene Symbol	Full Gene Name	Forward/Reverse Primer Sequence
CD137	Tumor necrosis factor receptor superfamily member 9	F: 5′-CGTGCAGAACTCCTGTGATAAC-3′ R: 5′-GTCCACCTATGCTGGAGAAGG-3′
TBX 1	T-box transcription factor 1	F: 5′-CTGTGGGACGAGTTCAATCAG-3′ R: 5′-TTGTCATCTACGGGCACAAAG-3′
TBX15	T-box transcription factor 15	F: 5′-CTCCGTTGAAGCCTTGATCGG-3′ R: 5′-AGACGCCAGGTCAGTGTGA-3′
UCP1	Uncoupling protein 1	F: 5′-AGGCTTCCAGTACCATTAGGT-3′ R: 5′-CTGAGTGAGGCAAAGCTGATTT-3′
TIMM44	Translocase of inner mitochondrial membrane 44	F: 5′-CTAGGCAGCGGAATCCAATTT-3′ R: 5′-GCAAGCCTGACAAAAACCCTTT-3′
NRG4	Neuregulin 4	F: 5′-CACGCTGCGAAGAGGTTTTTC-3′ R: 5′-CGCGATGGTAAGAGTGAGGA-3′
LPL	Lipoprotein lipase	F: 5′-GGGAGTTTGGCTCCAGAGTTT-3′ R: 5′-TGTGTCTTCAGGGGTCCTTAG-3′
ADIPO	Adiponectin	F: 5′-CGGCAGCACTGGCAAGTT-3′ R: 5′-CCGTGATGTGGTAAGAGAAGTAGTAGA-3′
ARP	Acidic-ribosomal protein	F: 5′-AAGCGCGTCCTGGCATTGTCT-3′ R: 5′-CCGCAGGGGCAGCAGTGGT-3′

## Abbreviations

AQP	Aquaporin
BAT	Brown adipose tissue
TAG	Triacylglycerol
UCP1	Uncoupling protein 1
WAT	White adipose tissue
